# Sports Nutrition for Optimal Athletic Performance and Health: Old, New and Future Perspectives

**DOI:** 10.1007/s40279-019-01224-4

**Published:** 2019-11-06

**Authors:** Lawrence L. Spriet

**Affiliations:** Human Health and Nutritional Sciences, University of Guelph, Guelph, ON N1G 2W1 Canada

This supplement examines several sports nutrition topics related to the optimisation of athletic performance and health. Some of the research areas have been examined for many years but are presently being examined with new methods and perspectives, while others are very new and some may be seen as approaches for the future. The authors have provided an excellent explanation of these new approaches and perspectives, as well as outlining where additional sports nutrition research is needed—especially with athletic populations and the long-term health of athletes. This collection of papers makes it very clear how important adequate nutrition is to the performance and well-being of athletes and how far reaching the negative effects of low energy availability can be, with these topics being discussed in five out of the eight papers in this supplement.

The Gatorade Sports Science Institute (GSSI) has been bringing sports nutrition and sports science researchers together for over 30 years to address and discuss many topics that relate to the health and performance of athletes. Since 2012, these meetings have been known as the GSSI Expert Panel. The latest meeting in March of 2019 was held to discuss old, new and future perspectives related to athlete nutritional, performance and health issues. Following the meeting, the authors summarized the recent work in their topic area, resulting in the manuscripts in this Sports Medicine supplement (the seventh in a series supported by GSSI).

The initial two papers highlight areas relevant to athletic performance that are difficult to study—does hypohydration really impair endurance performance [1] and what causes muscle cramping [2]? In the former situation, how does the experimenter effectively blind the research subject from knowing their hydration status? In addition, inducing hypohydration can be uncomfortable and unfamiliar to the subjects, with both problems potentially leading to performance decrements that are unrelated to hypohydration. The authors discuss recent attempts to rectify these problems using blinded hydration methods and conclude that hypohydration of ~ 2–3% body mass decreases endurance cycling performance in the heat, at least when no/little fluid is ingested [1]. In the muscle cramping situation, the authors have carefully described that water and salt balance are involved in cramping, using data from early studies in industrial settings with many subjects and also more recent work with smaller numbers of athletes [2]. In other situations, however, sustained abnormal spinal reflex activity seems to be the cause. Since no laboratory experimental models appear to be applicable to whole body exercise situations where muscle cramps occur, the authors argue that a single strategy for prevention or treatment will not be found.

In the next paper, De Souza et al. [3] provide a brief overview of the Female Athlete Triad and an update on the current thinking regarding energy availability. They also discuss the available literature relevant to a similar syndrome in males that is referred to as the Male Athlete Triad. To date, it appears that the energetic, reproductive and bone systems in men are more resilient to the effects of low energy availability compared to those of women, requiring more severe perturbations before alterations are observed. In addition, recovery of the hypothalamic pituitary gonadal axis occurs more quickly in men than in women. However, far more research with males experiencing low energy availability is needed.

The paper on nutrition and athlete bone health [4] also stresses the need for more athlete-specific research, especially as it relates to longer-term bone health (e.g., risk of osteopenia and osteoporosis) and shorter-term risk of bony injuries. Bone is a nutritionally modified tissue and generally benefits from weight-bearing activities, although not all athletes engage in weight-bearing sports. While nutritional requirements to support bone health may not be different between athletes and the general population, the authors highlight situations that may be relevant for athletes, including low energy availability, low carbohydrate availability, protein intake, vitamin D intake and dermal calcium and sodium losses. The paper by Walsh [5] outlines a new perspective on nutrition and athlete health to better understand how sick an athlete will become when they get an infection. This paradigm includes the concepts of immune resistance (destroying microbes) and immune tolerance (dampening defence but controlling infection to a non-damaging level). It also suggests that research efforts on nutritional supplements that may provide immunological tolerance and reduce the infection burden in athletes are needed.

The paper on nutrition and health at altitude [6] has been written by several scientists renowned for their work examining strategies to enhance adaptation, improve performance and maintain health in athletes living and training at low-to-moderate altitudes (1600–2400 m). Much of the existing altitude research was conducted at high to extreme altitudes (> 3000 m) and not the lower altitudes that athletes typically train at. While the authors highlight several nutritional issues that must be monitored at altitude, they stress that special attention must be given to the possibility of poor energy availability and increased iron requirements limiting the adaptations to altitude. Also, to deal with the possibility of increased oxidative stress at altitude, foods rich in antioxidants are recommended rather than high-dose antioxidant supplements.

The final two papers examine approaches to athlete nutrition and performance that might be called futuristic. The examination of blood test data, as a physiological profiling and monitoring tool, is becoming more routinely used in professional and elite high-performance athletes [7]. Much useful information can be obtained from blood tests, including the identification of iron, vitamin and energy deficiency, the identification of oxidative stress and inflammation status and the characteristics of red blood cell populations. Such data can be used to identify the effectiveness of training interventions, nutritional strategies and training load tolerance. The authors discuss perspectives, limitations and recommendations for sports science and sports medicine practitioners, who may use athlete blood profiling and monitoring for nutrition and performance purposes. In the final paper, Joyner [8] discusses the physiological determinants of human endurance performance, maximal oxygen uptake, the lactate threshold and running economy or effciency. He examines the genetics of endurance performance, as many of us may assume that individual differences in our genetic endowment would account for differences in endurance performance. However, he concludes that at present, interindividual differences in DNA sequence explain only a small fraction of the physiology underpinning sports performance.

The papers of this supplement have identified several areas of sports nutrition research that need to be studied or restudied to optimize sports performance and health for athletes. While we study groups of athletes, it is also clear that there are large individual variations between athletes and that we lack research in many areas for female athletes and in some cases male athletes. It is hoped that these papers have provided interesting perspectives on old, new and future areas of sports nutrition research and will spur additional research in these areas.
